# The blood-brain barrier disruption after syncope: a dynamic contrast-enhanced magnetic resonance imaging study: A case report

**DOI:** 10.1097/MD.0000000000028258

**Published:** 2021-12-17

**Authors:** Hyungkyu Huh, Eun-Hee Lee, Sung Suk Oh, Jong-Hoon Kim, Young Beom Seo, Yoo Jin Choo, Juyoung Park, Min Cheol Chang

**Affiliations:** a Medical Interdisciplinary Team, Medical Device Development Center, Daegu-Gyeongbuk Medical Innovation Foundation, Daegu, Korea; b Department of Neurosurgery, College of Medicine, Yeungnam University, Namku, Daegu, Republic of Korea; c Department of High-tech medical device, Gachon University, Seongnam, Republic of Korea; d SonoTx, Seongnam, Republic of Korea; e Department of Physical Medicine & Rehabilitation, College of Medicine, Yeungnam University, Namku, Daegu, Republic of Korea.

**Keywords:** blood-brain barrier, brain damage, hypoxia, magnetic resonance imaging, syncope

## Abstract

**Rationale:**

Using dynamic contrast-enhanced magnetic resonance imaging (DCE-MRI), we demonstrated blood-brain barrier (BBB) disruption following syncope.

**Patient concerns:**

A 45-year-old man experienced syncope with a chief complaint of syncope (duration: 1 minutes), 1 day before visiting a university hospital for examination. He had no history of medical problems and was not taking any medications. This episode was the first in his lifetime.

**Diagnoses:**

After syncope, the patient did not have any illnesses or symptoms, such as headache, cognitive deficits, or somnolence.

**Interventions:**

Cardiac evaluation did not reveal any abnormal findings. In addition, in conventional brain and chest computed tomography and brain MRI, no abnormal lesions were observed.

**Outcomes:**

DCE-MRI of the patient showed bright blue colored lines within the sulci throughout the cerebral cortex. The regions of interest, including bright blue colored lines, had significantly higher K_trans_ values (6.86 times higher) than those in healthy control participants. These findings are indicative of BBB disruption of the vessels in the sulci.

**Lessons:**

Using DCE-MRI, we demonstrated BBB disruption following syncope. DCE-MRI is a useful tool for the detection of BBB disruption following syncope.

## 1 Introduction

Syncope is an abrupt and transient loss of consciousness, which is induced by a drop in blood flow to the brain due to low blood pressure.^[1]^ There are various causes of syncope, including reflex-mediated syncope, cardiac, drug-induced, psychiatric syncope, orthostatic hypotension, hypoglycemia, and cerebral ischemia.^[1]^ Of these, reflex-mediated syncope is the most common.^[2]^ Although syncope is short, impaired blood supply during syncope reduces the delivery of oxygen and other essential nutrients, such as glucose, to the brain.^[3]^ We believe that this may damage brain structures and impair brain function. It has been reported that recurrent syncope episodes can lead to impairment of short-term memory.^[4]^ However, to date, the exact evidence of structural brain damage after syncope has not been reported.

Dynamic contrast-enhanced magnetic resonance imaging (DCE-MRI) is a noninvasive perfusion MRI technique that enables evaluation of damage to the microcirculatory structure and dysfunction of the brain-brain barrier (BBB).^[5–8]^ Several previous DCE-MRI studies have shown BBB disruption in various neurological disorders, including stroke, traumatic brain injury, dementia, mild cognitive impairment, and brain tumors.^[5–8]^

In this case report, we demonstrate BBB disruption after syncope using DCE-MRI.

## 2 Case presentation

A 45-year-old man (occupation: medical doctor) visited a university hospital with a chief complaint of fainting 1 day back. He had no history of medical problems and was not taking any medications. He had hiked 4 to 5 times a week for 2 years. Syncope occurred while hiking on a cold winter night (temperature: −8°C). He fainted while resting for a while in a standing position after hiking at a fast speed for an hour. The witness reported that he lost consciousness for about 1 minutes, and no head strike or seizure-like activity was observed. Upon awakening from syncope, the patient had no syncope recollection. After syncope, the patient did not have any illnesses or symptoms, such as headache, cognitive deficits, or somnolence. He had no prior episodes of syncope or fainting.

At the time of examination (the day after the syncope), his blood pressure was 122/77 mm Hg, and his resting heart rate was 75 beats per minute. Electrocardiogram, 24 hours ambulatory electrocardiogram monitoring, echocardiogram, tilt table test, and exercise tolerance test showed no abnormal findings. In addition, in conventional brain and chest computed tomography and brain MRI, no abnormalities were observed. All blood test results, such as electrolyte level, hemoglobin level, erythrocyte sedimentation rate, and C-reactive protein level, were normal. In the physical examination, he did not show any neurological symptoms, including motor, sensory, or cognitive deficits (Mini-Mental State Exam: full marks, no patient's subjective symptoms). The deep tendon reflex was normoactive in all the extremities. Based on the patient's history and examination results, the cardiologist diagnosed the patient with transient loss of consciousness due to syncope. In addition, considering that syncope occurred during excessive physical activity at cold temperatures, the possibility of vasovagal syncope was thought to be high.

DCE scans were acquired using a 3T system (Skyra, Siemens Healthcare, Erlangen, Germany). In addition to the patient, a DCE scan was obtained from a healthy control participant (42-year-old man) who volunteered for the study. Seven pre-contrast sets of DCE-MRI (echo time= 1.92 ms, repetition time= 5.46 ms, the field of view = 230 × 135 mm^2^, matrix size of 256 × 150, and slice thickness of 3 mm), followed by an additional 114 sets under the intravenous injection of contrast agent were imaged in axial view. The permeability (K_trans_) of the mid-slice was calculated using the Patlak model.^[9]^


$$C_{t}(t)=K_{trans}\int_{o}^{t}C_{p}(\tau )d\tau +V_{p}\cdot C_{p}(t)$$

**Figure 1:**
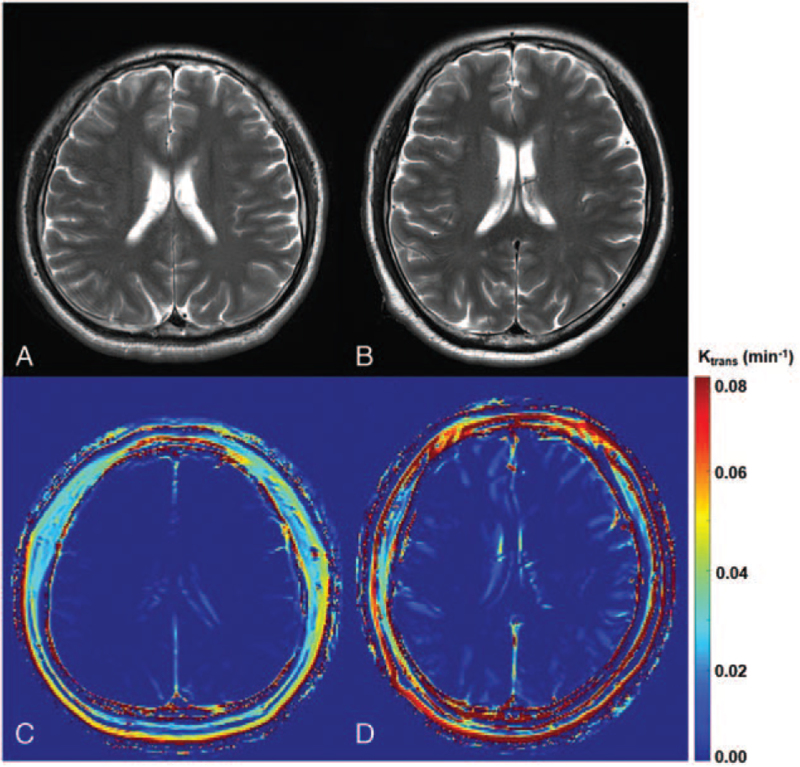
T2-weighted magnetic resonance imaging (MRI) and K_trans_ map of dynamic contrast-enhanced MRI of the healthy control participant (the 42-year-old man) and the patient (the 45-year-old man). (A) The T2-weighted MRI of the healthy control participant and (B) that of the patient show no abnormal finding. Ten round regions of interest (5 on each hemisphere) are depicted on the K_trans_ map of dynamic contrast-enhanced (DCE)-MRI of the healthy control participant and the patient (dotted circles in Figure c). (C) K_trans_ map of DCE-MRI of the healthy control participant shows no abnormal finding; however, (D) K_trans_ map of DCE-MRI of the patients shows bright blue colored lines throughout the overall sulcus.

Here, t indicates the time step, τ indicates the variable of integration, V_p_ indicates the volume of plasma, and C_t_ (t) and C_p_ (t) indicate the temporal variation of the concentration of the contrast agent in the tissue and plasma, respectively. A circular region of interest (ROI) with an inner diameter of 1 to 2 cm was located at the superior sagittal sinus, which has higher consistency and plausibility compared to the internal carotid artery or the arterial vessel closest to the lesion.^[10]^ In the axial image where the body and crus of the fornix were presented, 10 round random ROIs of 2 cm diameter (dotted circles in Fig. 1) were selected (5 in each hemisphere) to quantify the overall averaged K_trans_ of the brain. ROIs were depicted, including sulci in which the bright blue colored lines (BBB disruption) are presented.

For the quantitative analysis, in each ROI of the healthy control participants and the patients, the T2 and K_trans_ values were measured. The differences in the measured values between the healthy controls and patients were compared using an independent *t*-test. Statistical significance was set at *P* < .05.

In the conventional MRIs of the healthy control and the patient, no lesions were observed (Fig. 1A, 1B). In addition, in the K_trans_ map of DCE MRI of healthy control participants, no specific abnormal findings were observed (Fig. 1C). However, in the patient, bright blue-colored lines were observed within the sulci throughout the cerebral cortex (Fig. 1D). These findings indicate disruption of the BBB of vessels in the overall cerebral sulci. In the quantitative analysis, the averaged T2 intensity (within 10 randomly selected ROIs) of healthy control and patient was 403.95 ± 24.91 (AU) and 404.50 ± 24.21 (AU), which was not statistically different (*P* = .9625). Here, the average K_trans_ of healthy controls within 10 randomly selected ROIs was 0.000156 ± 0.000099 (minutes^−1^) and 0.001067 ± 0.000806 (minutes^−1^) for the patient. The average K_trans_ of the patient was 6.86 times higher than that of the healthy controls, and the difference was statistically significant (*P* = .003409).

## 3 Discussion

In the current report, using DCE-MRI, we investigated BBB disruption following syncope. On DCE-MRI of the patient, bright-blue colored lines were observed in the overall sulci, although conventional MRI did not show any remarkable lesions. These findings indicate BBB disruption. Considering that the red, light green, and bright colors in DCE-MRI indicate severe, moderate, and mild BBB disruption, respectively, the degree of BBB disruption in our patient is thought to be mild.^[11]^

Additionally, the values of average K_trans_ in the ROIs depicted, including the bright blue colored lines, were significantly higher in the patient than in the normal control. K_trans_ is one of the most frequently used values for assessing BBB disruption, which indicates volume transfer contrast from the plasma into the extracellular extravascular space.^[9]^ Therefore, BBB disruption increased K_trans_. The increased value of K_trans_ in our patient is a quantitative finding confirming BBB disruption.

BBB disruption occurs in the vessels of the sulci throughout the cerebral cortex. This phenomenon appears to be correlated with the fact that the brain cortex is particularly vulnerable to hypoxic conditions.^[12]^ The insufficient supply of oxygen and glucose at the time of syncope inhibits ATP production. Reduced ATP levels in the brain impair the function of Na^+^-K^+^-ATPase and Ca^2+^-ATPase activity in the cellular membrane.^[13,14]^ This results in the accumulation of Na^+^ in the endothelial cells of the BBB, which leads to endothelial cell swelling and BBB breakdown.^[13,14]^

Vasovagal syncope is the most common type of reflex syncope induced by a sudden drop in blood pressure.^[15]^ It causes a drop in blood flow in the brain, resulting in an abrupt and transient loss of consciousness. The cause of vasovagal syncope is unclear. However, it has been suggested that vasovagal reaction is an exaggerated adaptive response for assisting hemostasis, such as lowering blood pressure and heart rate to reduce the bleeding volume, in the setting of physical trauma.^[15]^ Vasovagal syncope occurs frequently, with a lifetime incidence of more than 30%.^[16]^ The etiology of vasovagal syncope is often unidentifiable; however, pain or emotional stress is known to be a frequent cause of vasovagal syncope.^[17]^ In addition, central hypovolemia from dehydration or upright posture increases the risk of vasovagal syncope.^[15]^ This state increases cardiac contractility, which can trigger mechanoreceptors in the cardiac ventricle that stimulate vagal afferents to the central nervous system.

Some previous studies reported that alterations in DCE-MRI parameters, such as K_trans_, were significantly correlated with poor cognitive performance following traumatic brain injury.^[18,19]^ We believe that BBB disruption can indirectly implicate microscopic injury to the brain parenchyma.^[9]^ We showed that syncope can result in BBB disruption. In our case, as the syncope duration was short, the degree of BBB disruption was mild, and specific neurological symptoms did not manifest. However, if the syncope duration is long or repetitive, the degree of BBB disruption can be moderate or severe, causing various neurological deficits.

## 4 Conclusion

In the current report, using DCE-MRI, we demonstrated BBB disruption following syncope. DCE-MRI is a useful tool for the detection of BBB disruption following syncope. This is the first report to show that BBB disruption occurs after syncope. However, our study is limited in that it was a case study. Further studies involving a larger number of participants are required.

## Author contributions

**Conceptualization:** Hyungkyu Huh, Eun-Hee Lee, Sung Suk Oh, Jong-Hoon Kim, Young Beom Seo, Yoo Jin Choo, Juyoung Park, Min Cheol Chang.

**Data curation:** Hyungkyu Huh, Eun-Hee Lee, Sung Suk Oh, Jong-Hoon Kim, Young Beom Seo, Yoo Jin Choo, Juyoung Park, Min Cheol Chang.

**Formal analysis:** Hyungkyu Huh.

**Investigation:** Hyungkyu Huh, Min Cheol Chang.

**Methodology:** Hyungkyu Huh, Eun-Hee Lee, Min Cheol Chang.

**Resources:** Hyungkyu Huh, Min Cheol Chang.

**Software:** Hyungkyu Huh.

**Supervision:** Juyoung Park, Min Cheol Chang.

**Validation:** Min Cheol Chang.

**Visualization:** Hyungkyu Huh, Min Cheol Chang.

**Writing – original draft:** Hyungkyu Huh, Eun-Hee Lee, Sung Suk Oh, Jong-Hoon Kim, Young Beom Seo, Yoo Jin Choo, Juyoung Park, Min Cheol Chang.

**Writing – review & editing:** Hyungkyu Huh, Eun-Hee Lee, Sung Suk Oh, Jong-Hoon Kim, Young Beom Seo, Yoo Jin Choo, Juyoung Park, Min Cheol Chang.
